# Empowering Health Care Actors to Contribute to the Implementation of Health Data Integration Platforms: Retrospective of the medEmotion Project

**DOI:** 10.2196/68083

**Published:** 2025-03-04

**Authors:** Marcel Parciak, Noëlla Pierlet, Liesbet M Peeters

**Affiliations:** 1 Biomedical Research Institute UHasselt - Hasselt University Diepenbeek Belgium; 2 Data Science Institute UHasselt - Hasselt University Hasselt Belgium; 3 Ziekenhuis Oost-Limburg Genk Belgium; 4 UHasselt - Hasselt University University Multiple Sclerosis Center Diepenbeek Belgium

**Keywords:** data science, health data integration, health data platform, real-world evidence, health care, health data, data, integration platforms, collaborative, platform, Belgium, Europe, personas, communication, health care providers, hospital-specific requirements, digital health

## Abstract

Health data integration platforms are vital to drive collaborative, interdisciplinary medical research projects. Developing such a platform requires input from different stakeholders. Managing these stakeholders and steering platform development is challenging, and misaligning the platform to the partners’ strategies might lead to a low acceptance of the final platform. We present the medEmotion project, a collaborative effort among 7 partners from health care, academia, and industry to develop a health data integration platform for the region of Limburg in Belgium. We focus on the development process and stakeholder engagement, aiming to give practical advice for similar future efforts based on our reflections on medEmotion. We introduce Personas to paraphrase different roles that stakeholders take and Demonstrators that summarize personas’ requirements with respect to the platform. Both the personas and the demonstrators serve 2 purposes. First, they are used to define technical requirements for the medEmotion platform. Second, they represent a communication vehicle that simplifies discussions among all stakeholders. Based on the personas and demonstrators, we present the medEmotion platform based on components from the Microsoft Azure cloud. The demonstrators are based on real-world use cases and showcase the utility of the platform. We reflect on the development process of medEmotion and distill takeaway messages that will be helpful for future projects. Investing in community building, stakeholder engagement, and education is vital to building an ecosystem for a health data integration platform. Instead of academic-led projects, the health care providers themselves ideally drive collaboration among health care providers. The providers are best positioned to address hospital-specific requirements, while academics take a neutral mediator role. This also includes the ideation phase, where it is vital to ensure the involvement of all stakeholders. Finally, balancing innovation with implementation is key to developing an innovative yet sustainable health data integration platform.

## Introduction

Accurate and well-formatted data are key to delivering high-quality health care and fueling medical research [1-3]. All health care actors acquire real-world data, defined as any health care-related information captured from the patient [4]. The volume, velocity, and variety of acquired data, however, raise challenges for data processing systems [5]. Data engineers work in interdisciplinary environments to ensure that users receive data in the right format, at the right time and place to generate real-world evidence [4]. Health data engineering is a complex and time-consuming task that cannot be managed without IT solutions tailored to environment-specific requirements [6]. Consequently, health data integration platforms are a hot topic in medical informatics research [7-11]. Health data integration commonly refers to finding relevant and rich patient information on time with appropriate interfaces [6]. In this paper, we present our design and development approach to building such a platform.

The medEmotion project was a collaborative initiative among 3 hospitals (Jessa Ziekenhuis, Noorderhart, and Ziekenhuis Oost-Limburg), 2 academic institutions (Hogeschool PXL, and UHasselt), and 2 industry partners (LRM, and BioVille) from the region of Limburg, Belgium. The primary objective of medEmotion was to establish a comprehensive data integration platform designed to effectively address a wide array of health data-related inquiries and challenges encountered by various stakeholders within the health care ecosystem. The medEmotion platform aggregates medical data from hospitals and general practitioners, personal health information collected through wearable devices, and environmental data. It enables researchers, health care professionals, and entrepreneurs to test innovative data-based concepts and generate real-world evidence. We built the medEmotion platform to be secure and compliant with legal standards to be fit for purpose for real-world health data. The platform enhances decision-making and stakeholder collaboration by facilitating comprehensive analysis of integrated real-world health data.

To guide and showcase the data integration platform developed in medEmotion, we defined 2 real-world use cases for distinct disease areas. First, with the Noorderhart hospital, we approach the multiple sclerosis data connect (MSDC) use case, focusing on the health care process of people with multiple sclerosis. MSDC is characterized by a relatively low total amount of patients just below a thousand where each patient is described with a high amount of diverse information in the form of structured clinical data, evoked potential time series data, magnetic resonance images, and para-clinical data, such as information collected during physiotherapy. We thus obtain a broad dataset that brings a distinct set of challenges from the second use case in medEmotion, Cardio. Developed in collaboration with Jessa Ziekenhuis and Ziekenhuis Oost-Limburg, we consider the postclinical workflows of cardiology patients. Cardio is characterized by a low variety and high velocity of data collected per patient. Next to a basic set of clinical variables, most data are acquired through wearable sensors. We thus have a data source that continuously generates new data points.

In this paper, we outline the medEmotion project, focusing on our chosen development approach. In particular, we describe a team of personas and a set of demonstrators. Personas are fictional stakeholders whose requirements and unique perspectives we implemented into the project. Demonstrators describe sets of functional requirements of the medEmotion platform aiming to solve real-world use cases. Each demonstrator’s requirements are affected by one or more personas.

Furthermore, we discuss our approach, combining personal reflections on our challenges during medEmotion. With these reflections, we reminisce about our experiences that we believe present common pitfalls of health care-related projects that handle real-world data. We aim to shed light on the tension between stakeholders from health care, academia, industry, and investment societies and give advice for future efforts.

The paper is structured as follows. We introduce the personas and the demonstrators of medEmotion alongside a summary of its implementation. Afterward, we discuss our development approach and the result. We end with distilling key takeaway messages from the medEmotion project.

## Personas

**Figure 1:**
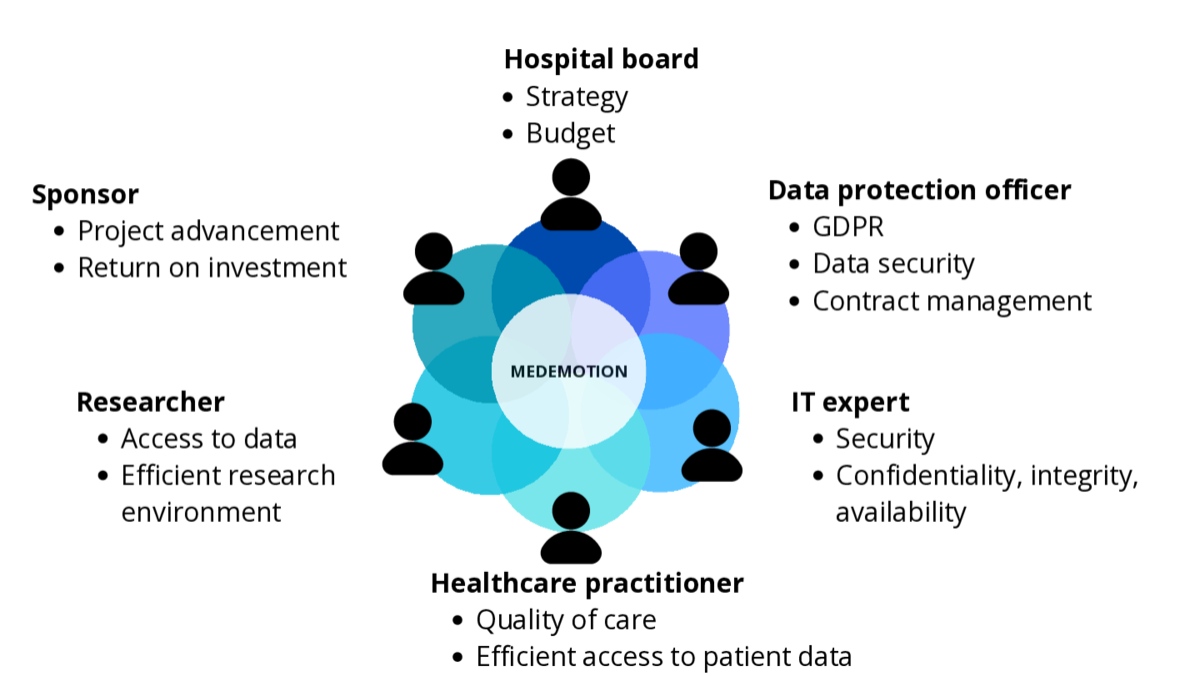
Personas involved in the different demonstrators, with some of their focus points indicated. GDPR: General Data Protection Regulation.

Stakeholder management is a core project management task that aligns the requirements and visions of stakeholders. A stakeholder may be part of the core project team or take a consultant role, advising the project team regularly. From our experience, most stakeholders combine multiple personas. Each stakeholder might take on a different “hat,” sharing their view and arguments in different, situation-specific ways to drive the project forward. Therefore, we define 6 personas that represent the different “hats” a stakeholder may represent in the following (Figure 1). The following personas are based on observations during medEmotion, we thus do not claim this list to be exhaustive. We think this list will serve as a starting point to identify personas in other projects.

### Data Protection Officer

The Data Protection Officer persona is concerned with keeping information up to date, private, secure, and traceable. These concerns can be enforced by law, such as the General Data Protection Regulation (GDPR) of the European Union, or can be intrinsic in the sense that keeping data well governed makes aspects such as data security and contract management easier. People who represent this persona are Data Protection Officers of hospitals, contract managers, or data stewards.

### Researcher

The Researcher persona is concerned with conducting research more efficiently or easily. In health data integration, conducting research typically means running experiments on real-world health data. Thus, the researcher aims to get easier access to health data, larger amounts of health data, more computational resources, or access to software tools that accelerate data processing. A common example of a researcher is a PhD student.

### Health Care Practitioner

The health care practitioner is concerned with delivering the best health care quality to the patient. To do so, they need recent, correct, and sufficient information about a patient while they consult with them or decide on future medical interventions. For example, clinicians and nurses represent this persona.

### IT Expert

The IT Expert persona is concerned with efficiently and reliably running the hospital information system (HIS). The IT-security aims of confidentiality, integrity, and availability are of major concern, which results in a more reluctant attitude toward new and unknown systems. The hospital’s chief information officer represents this persona.

### Hospital Board Member

The hospital board member persona is concerned with the strategic advancement of the hospital within the resources available. As such, this persona will wage strategic and long-term goals, such as participating in international research studies, against short-term available resources, such as the workforce and budget of a hospital. For example, a hospital’s chief executive officer represents this persona.

### Sponsor

The Sponsor persona is concerned with the success of the investment. In a research project such as medEmotion, this includes ensuring that project resources are well-spent and securing potential return on investments about the project results. This persona is represented by project sponsors, such as venture capital, pharma, or insurance companies.

During medEmotion, we found that defining personas and assigning them to our stakeholders made discussions more transparent and efficient. The set of people participating in discussions and meetings changes frequently during a project. A list of predefined personas allowed us to ensure we included all distinct perspectives to decide on the next steps. In particular, we assigned these personas to demonstrators to gain well-structured functional requirements, which we will list in the following section.

## Demonstrators

We defined a series of real-world demonstrators to facilitate the requirement engineering process. Each demonstrator represents a bundle of related technical requirements that include the unique perspectives of all personas involved. In particular, our demonstrators are disease-agnostic and can be applied to any real-world dataset. By contrast, the use cases we used in medEmotion describe higher-level and disease-specific requirements that affect multiple demonstrators. We present a short description of each defined demonstrator, and the personas involved.

### Orchestration of Complex Pipelines

In biomedical research projects, (para-)clinical data must be processed through complex data integration steps. Researchers often implement these steps ad hoc using different programming languages (eg, Python [Python software foundation], R [R foundation], MATLAB [MathWorks]), and software frameworks (eg, Torch [PyTorch Foundation], Pandas [Pandas project], dplyr [posit]) or reuse existing implementations as black boxes [12]. The resulting software code is complex and difficult to automate, orchestrate, and reproduce, pushing it out of reach for IT experts. Further, data protection officers see a lack of in-depth documentation, essentially losing real-world data due to missing knowledge. With this demonstrator, we showcase the platform’s capabilities to orchestrate complex data integration pipelines automatically.

### Private Research Environments

Access to real-world data greatly increases the efficiency of exploratory research tasks. With strict privacy regulations such as the GDPR of the European Union or the HIPAA (Health Insurance Portability and Accountability Act) of the United States, data access is often achieved by sharing datasets after bilateral contracts between the data custodians (eg, hospitals) and universities are set in place [13]. This process, however, is slow, complex, and lacks transparency, creating tension between researchers, Data Protection Officers, and IT experts. Researchers need to deal with tedious administration tasks, while data privacy officers and IT experts are concerned with keeping sensitive data secure. With this demonstrator, we showcase the platform’s ability to create private research environments that allow researchers to access data, tooling, and compute resources while keeping real-world health data secure.

### Observational Health Data Sciences and Informatics Observational Medical Outcomes Partnership Common Data Model

Transnational medical research can be facilitated with internationally standardized data schemas, such as the Observational Medical Outcomes Partnership (OMOP) common data model (CDM) developed by the Observational Health Data Sciences and Informatics (OHDSI) research community. Transforming health care records to OMOP is a highly operational task, enabling data custodians to participate in federated, transnational research projects without sharing sensitive datasets [14]. Transforming and maintaining data structured according to the OMOP CDM is desirable for researchers and hospital board members, as it allows for simple access, privacy perseverance, and increased hospital visibility in the academic landscape. With this demonstrator, we showcase the platform’s ability to facilitate transforming data to and maintaining data in the OMOP CDM, including running a suite of software tools developed by the OHDSI community.

### Visualization of Integrated Datasets to Support Care

Providing high-quality health care necessitates large amounts of integrated, patient-centric data that facilitate informed decision-making. Analyzing these extensive amounts of patient data and gathering insights on the evolution of the patient’s medical conditions over time in a few minutes is only possible with data-rich visualizations [15]. The clinical decision-making process remains health care practitioner–specific as well as multidisciplinary. Hence, visualizations must be adaptable per individual and still sharable within a multidisciplinary team to stay helpful. Privacy remains essential in the eyes of Data Protection Officers, hence, sophisticated methods to share information according to therapeutic relationships between health care practitioners and patients are needed. With this demonstrator, we showcase the platform’s ability to facilitate online visualizations of rich datasets that can be tailored to individual needs and shared among multidisciplinary teams.

### Data Ingestion From Hospital Data

The primary sources of real-world data meant to generate real-world evidence are HISs. Even for clinical studies that collect data with study-specific case report forms, HIS data helps provide important context information, such as demographics or comorbidities [1]. Due to the value of HIS data, one of the sponsor’s main targets is integrating such data into the platform. This real-world data is highly sensitive and the prime target of data protection measures set up by data protection officers and IT experts. Researchers aiming to access the data naturally clash with data protection measures, as they tend to over-request data. With this demonstrator, we showcase the platform’s ability to ingest data from multiple pseudonymized data sources.

### Data Ingestion From Devices

With eHealth solutions on the rise, data integration platforms receive more interest from sponsors to include data collected outside the traditional boundaries such as the hospital or the practice. Patients collect data themselves, either actively using health apps or passively using wearable devices [16], both using lifestyle and software-as-medical device apps [17]. Providing richer datasets for clinicians and researchers, IT experts face challenging data integration tasks of high volume and erroneous-prone data streams. Of course, these data streams are sensitive and thus are a concern of Data Protection Officers. With this demonstrator, we showcase the platform’s ability to include data streams from home monitoring devices that are not part of traditional hospital information systems.

### Connection With High-Performance Computing Centers

High-performance computing (HPC) enables researchers to use machine-learning approaches [18]. Those can ramp up costs quickly when acquired from public cloud providers such as Microsoft Azure, Google Cloud, or Amazon Web Services. Many academic institutions operate HPC centers to meet the demand for HPC. To keep research projects using machine learning feasible, data transfer to and from academic HPC centers needs to be simple while retaining IT security standards set by data protection officers. With this demonstrator, we showcase the platform’s ability to transform data between the Vlaams Supercomputer Center, an HPC center operated by and for the 5 Flemish universities.

### Summary

The demonstrators guided discussions and the implementation of the medEmotion platform. Based on the personas, we could identify which stakeholders to include in discussions. Hence, we were able to have more ad-hoc discussions in smaller groups, replacing large and time-consuming meetings to discuss the entirety of the platform components. Combining personas and demonstrators allowed for a rapid implementation process, which we summarize in the following.

## Implementation

We implemented the medEmotion platform with an industry partner using Microsoft’s Azure Cloud Platform components. The platform’s architecture foresees a general environment and multiple use case-specific data silos. We find modules and tools in a general environment that handle pseudonymized, cleaned, and integrated data. In the hospital-specific data silos, we find modules and tools to preprocess datasets either for the general environment or for hospital-specific projects. The implementation process was guided and managed by using case-specific task forces that met at regular intervals. Each task force consisted of relevant stakeholders from multiple partners that covered all personas as described above. Each task force meeting was attended by a member of the medEmotion core team which summarized the discussions and extracted feedback, suggestions, and additional requirements that were reported to the implementing industry partner.

To ensure data privacy, data from different source systems is pseudonymized with source-specific keys on entering the data platform, such that the data in the medEmotion platform cannot be linked without additional effort. The platform retains encrypted quasi-identifiers, information irrelevant to answer clinical questions that potentially reveal the identity of a patient, to allow for ad-hoc record linkage if needed. The encrypted identifiers are locked away in a protected zone in the platform, and the keys to decrypt the identifiers are locked away in Azure Key Vaults. Next to the security guarantees provided by Microsoft Azure with respect to the Key Vaults, the medEmotion platform could also be implemented with on-premises Key Vaults, granting full control to data custodians with respect to the decryption keys used to link data that stems from their systems. In our proof-of-concept implementation, we used a Key Vault operated within the Microsoft Azure public cloud.

The figure shows components from 3 environments: On-premises (eg, a partner hospital), the Azure cloud, and External, which shows users outside the platform. Where applicable, we name the Microsoft Azure product used written in bold. The arrows indicate data flows, starting with the data sources on the left and flowing to the data users on the right.

The demonstrators were implemented exclusively with public or mock datasets, that was synthetically generated data modeled after real-world datasets. We created a mock data source following health level-7 FHIR (Fast Healthcare Interoperability Resources) [19] for the data ingestion demonstrators. The Azure FHIR Store is a natural fit for this data source. Further, an industry partner that offers wearable eHealth devices supplied us with a mock data stream for our eHealth device demonstrator. We generally use a decentralized lightweight pseudonymization service that runs on-premise at a hospital. This service polls for and pulls data in scheduled intervals from the sources, pseudonymizes it, and ingests it into the platform using Azure Data Factory.

To showcase the OHDSI OMOP CDM demonstrator, we generated a mock dataset based on a multiple sclerosis–specific data acquisition system, which we transformed into the OMOP CDM format. With this data, we operationalized OHDSI tools such as ATLAS to test the transformed data [14].

**Figure 2:**
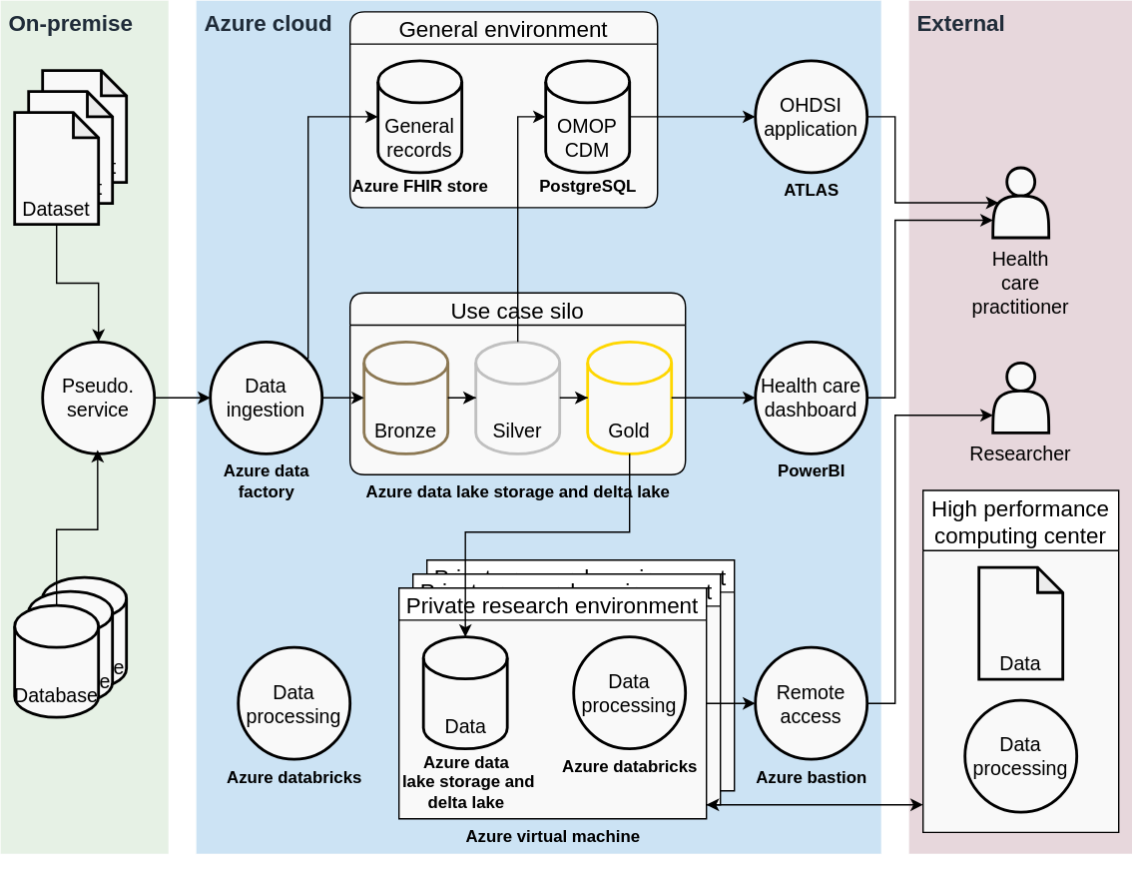
A high-level overview of the medEmotion platform implementation. The figure shows components from 3 environments: On-premises (eg, a partner hospital), the Azure cloud, and External, which shows users outside the platform. Where applicable, we name the Microsoft Azure product used written in bold. The arrows indicate data flows, starting with the data sources on the left and flowing to the data users on the right. CDM: common data model; FHIR: Fast Healthcare Interoperability Resources; OHDSI: Observational Health Data Sciences and Informatics; OMOP: Observational Medical Outcomes Partnership.

We used the datasets described by Yperman et al [18] for the remaining demonstrators. We use the Yperman et al [20] open-access dataset and an open-access magnetic resonance image dataset from the MICCAI [21] 2016 challenge. We use these datasets to showcase the orchestration of complex pipelines with Azure Databricks, as this enables our existing preprocessing scripts to be automated, orchestrated, and monitored. We encapsulate Azure Databricks in Azure Virtual Machines, where we block all connections to public networks (such as the web) to showcase private research environments. With the same tools but less stringent blocking rules (ie, we allow connection to selected HPC centers using virtual private network connections), we enable the use of HPC resources from within the platform. Finally, we use Microsoft’s PowerBI to create dashboards per health care practitioner’s requirements. In particular, we built an online, data-rich dashboard that respects therapeutic relationships (Figure 2).

## Limitations

We combined personas and demonstrators to effectively steer the development and implementation of the medEmotion platform, resulting in a feature-rich yet adaptable solution. The use of mock-up datasets has proven particularly useful to accelerate development, as access to patient-level data remains a time-consuming process. While we focused on showcasing the technical feasibility of a data integration platform, the support for organizational tasks is currently missing from the medEmotion platform. The medEmotion platform does not enforce any data governance rules and does not offer any user-friendly user interfaces to manage data access rules. We put these organizational aspects out of scope, and we note that tackling these questions is essential to implementing the medEmotion platform in practice. During medEmotion, we focused on a proof-of-concept data integration platform. Hence, we did not tackle technical aspects to make the medEmotion platform production-ready, such as data redundancy or a multitier architecture. We acknowledge that these questions are important to answer in production environments and note that they are limited by a hospital’s strategy, budget, and IT resources. Therefore, we do not present an answer to these aspects as they are highly hospital-specific and ultimately need to be decided per hospital.

In the remainder of this paper, we review the development process of the medEmotion platform.

## Discussion

One of the key successes of the medEmotion project was its pioneering role in bringing together a diverse group of partner hospitals, academic institutions, and industry stakeholders, for the first time in a concrete, collaborative effort. This collaboration allowed us to gain invaluable insights into the distinct needs and concerns of various stakeholders, paraphrased as personas as mentioned before, ranging from data protection and privacy issues to the practical demands of health care practitioners. Through this partnership, we also learned a great deal about the organizational challenges that remain unaddressed in multistakeholder health data initiatives. To give 2 examples, we have seen that hospital-specific IT strategies vary significantly. While one hospital works within a cloud-based IT infrastructure, another prohibits using cloud-based components in favor of an exclusively on-premise infrastructure. The same can be said for data management strategies. Where one hospital aims to keep data per department in distinct and tailored information systems, another aims to centralize as much information as possible into a single monolithic system. Personas apply these strategies in discussions but do not necessarily make them explicit. We believe that learning these implicit factors is vital to successful initiatives. Discussions about implementing a health data integration platform gave in-depth insights into hospital-specific data flows. We outlined how patient information flows into the hospital information system, an important exercise that improved mutual understanding. Importantly, beyond the technical knowledge gained, this project fostered personal connections and mutual trust among individuals representing different areas of expertise and roles within their organizations. This familiarity and trust built over time are essential for future interdisciplinary projects.

Clarifying technical questions using our demonstrators was pivotal in making abstract concepts more tangible for stakeholders. Much like trying to build a car in a world where cars do not yet exist, stakeholders initially struggled to articulate their needs without a clear vision of what the final product could look like. In such a setting, any implementation process will fail, as the result cannot fit the stakeholders’ unarticulated vision. The demonstrators provided a concrete basis for discussions, allowing individuals without extensive technical backgrounds to actively engage and provide meaningful feedback. This practical approach significantly improved communication between the partners and helped bridge the gap between different personas. As such, we were able to develop and implement an architecture consisting of individual modules that both satisfied our demonstrators and generated general approval from the stakeholders.

Despite the many successes of the medEmotion project, several frustrations and challenges emerged throughout its course. Looking back, there were decisions and approaches that, with the benefit of hindsight, could have been handled differently to overcome obstacles more effectively. In this section, these key frustrations are summarized.

### Effective Communication and Collaboration in a Fluid and Diverse Team Proved Extremely Challenging

With members frequently joining and leaving, maintaining continuity of knowledge was difficult, leading to disruptions in collaboration. The large and diverse group of individuals, each bringing distinct backgrounds, interests, and expertise, made aligning perspectives and ensuring consistent communication even more complex. This constant flux created coordination barriers, making it hard to sustain productive collaboration and slowing down decision-making processes.

### Conflicting Priorities Among Key Stakeholders Created Significant Challenges in Aligning Goals and Decision-Making

Some stakeholders were primarily focused on return on investment and long-term strategic gains, while others were more concerned with its innovative potential and practical usability. This disconnect led to frequent misalignments, where decisions made often overlooked the technical needs or the full potential of the platform, or led to less effective outcomes as critical insights were missing in the decision-making process. As a result, in some cases, the project appeared to be driven more by academic interests rather than the practical needs of the health care partners. While the platform seemed promising on paper, there was a disconnect between the project’s goals and the real-world priorities of the hospitals. For some hospitals, the platform was unsolicited and not aligned with their internal strategic plans, leading to doubts about its relevance. Additionally, one of the partner hospitals already had a similar platform in place, reducing the added value of the new system.

### The Mismatch Between the Data Demands of Researchers and the Hospitals’ Reluctance to Share Sensitive Data for Secondary use Complicated the Project

From the hospital’s perspective, the process of preparing data cleaning, anonymizing, and transforming it represents a costly and time-consuming task. There is limited immediate benefit while they expose themselves to significant risks concerning the GDPR. Real-world data is highly sensitive and requires highly secured IT environments, which common academic research projects fail to consider. These conflicting needs slowed progress and exposed gaps in the project’s ability to balance the expectations of different stakeholders, especially regarding the complexities of data sharing.

### The Project Faced Significant Hurdles Related to Funding and Intellectual Property

The traditional “project-to-project” funding model limited the potential for long-term commitment. Without a clear financial roadmap beyond the initial phase, maintaining stakeholder engagement became increasingly difficult. At the same time, intellectual property (IP) issues arose as partners contributed in different ways, intellectually, financially, or through providing real-world use cases, making it complicated to define ownership. The absence of early, transparent IP agreements led to terms of usage that were unacceptable for some partners, limiting the potential for certain project components to be used effectively post the project. This further hindered the scalability and long-term success of the initiative.

### Balancing the Openness of Collaboration With the Security and Operational Needs of Health Care Organizations Remains a Critical Challenge

Investing in open science can offer significant advantages in collaborative projects. Open-source software fosters transparency and inclusivity by allowing all partners to access, modify, and contribute to the project. This transparency builds trust among stakeholders as everyone can see how their contributions are used and built upon. Open science also reduces barriers to entry, enabling partners who may lack financial resources to still contribute valuable intellectual input, leveling the playing field, and encouraging broader collaboration. Open-source environments can also drive faster innovation, as contributors freely share ideas, experiment, and improve each other’s work. This collaborative approach often accelerates development and produces more robust, well-tested solutions. Furthermore, open-source projects benefit from collective maintenance and support by the community, making them more sustainable in the long term, even after the original funding or key contributors move on. However, there are pitfalls to consider. Open-source solutions, while valuable in academic settings, often conflict with hospital policies that prioritize stringent security measures and professional support. Hospitals may require more controlled environments to meet compliance and data privacy regulations, which may not always align with open-source principles.

### Rapid Scale-Up and Professionalization Impacted the Balance Between Innovation and Implementation

A small group of researchers, who had initially focused on exploring and developing innovative solutions, had to quickly shift from this exploratory work to handling operational tasks as the project grew. This sudden change required them to manage the day-to-day implementation and ensure compliance with legal and technical requirements, leaving less time for creative research and experimentation. As a result, the researchers experienced a drop in motivation, finding themselves in presentations that felt more like sales pitches than discussions of research results. With no room for experimental approaches, the rapid scale-up and professionalization of the project brought innovation to a halt, a frustrating experience for those with a research mindset, who thrive on flexibility and exploration.

### Focusing on Developing a Proof-of-Concept Limited the Production-Readiness of the Platform

The development of the medEmotion platform was bounded by time and resources. Initially focused on providing a minimal viable product, we approached implementation in little increments to provide quick and tangible parts of the platform. While this allowed for rapid development, our approach omitted important elements to make the platform ready for production. The use of mock datasets allowed us to push data privacy issues to the end, the cloud-based development left sensitive data flows from the hospital to the platform untested, and the rapid implementation start left us with no comparison metrics to assess the medEmotion platform user satisfaction. All these limitations persist and now need to be tackled post implementation, increasing the difficulty of bringing the medEmotion platform into production. As such, the medEmotion platform currently remains in a proof-of-concept state, not being implemented at any of our partner hospitals.

### Takeaway Messages

We believe we are neither the first nor the last researchers to experience these or similar frustrations. Therefore, we share our experiences as takeaway messages in the following. Based on our experience, we aim to offer practical advice that can help guide future multistakeholder collaborative initiatives in health care and data integration.

### Invest in Community Building, Stakeholder Engagement, and Education

Creating opportunities for stakeholders to get to know each other, both at the organizational level and on a personal level, is crucial for building trust and understanding. From an organizational perspective, this helps clarify the distinct needs of each stakeholder group and ensures that strategic goals are aligned toward a shared objective where value can be realized for everyone, even if the “return on investment” may look different for each party. On a more personal level, trust is not just an institutional concept; it is a feeling that grows between individuals and thus takes time. Providing opportunities for stakeholders to form personal connections through trustworthy relationships can significantly enhance collaboration. Organizing networking events, workshops, or joint educational sessions can help foster these connections and build a strong foundation of mutual trust and respect, which will pay dividends throughout the project.

### Collaboration Among Health Care Providers Is Ideally Driven by the Health Care Providers Themselves

The health care providers are in the best position to proactively share expertise and requests for assistance based on their unique needs. Academic partners can play a key role in facilitating partnerships as neutral mediators, helping to bridge gaps between institutions. While commercial partners bring valuable expertise, maintaining a focus on shared goals and minimizing potential conflicts of interest can be achieved by ensuring that collaboration remains centered on the needs and priorities of health care providers. However, when it comes to implementation, there are additional challenges that need to be addressed, particularly within health care settings. Health care providers must establish secure data transfer protocols and ensure that patients are informed about how their data will be shared.

### Ensure All Stakeholders Are Involved in the “Ideation Phase”

It is essential to spend adequate time understanding the problem and the real needs and expectations of all stakeholders and future users. One effective approach is to use qualitative research methodologies, such as semi-structured interviews and focus groups, before moving into the IT development phase. For example, methodologies from the newly launched educational program “System and Process Innovation in Healthcare” at Hasselt University could be leveraged to guide this process. In addition, involving legal departments early, particularly those within health care organizations is vital for ensuring compliance and managing risks effectively. It’s also critical to align IP ownership and a long-term funding strategy at the outset. Establishing clear IP agreements and a roadmap for continued financial support ensures the sustainability of the platform beyond the initial project phase. Finally, it is equally important to sanity-check technical plans with experts to guarantee feasibility, especially given the usual constraints of a fixed budget. Early input from all relevant parties helps prevent misalignments and avoids costly revisions later in the project.

### Balance Innovation With Implementation

To ensure long-term success, it is important to strike a balance between pilot projects that foster innovation and the implementation of existing, proven solutions. These are fundamentally different activities, requiring distinct profiles and skill sets. Innovation thrives when motivated partners are eager to try new methods and solutions. This kind of exploratory work benefits from a small, focused group of individuals with an innovation and research mindset who can freely experiment and iterate based on the real-world needs of partners. In this context, both parties gain from the trial-and-error process that allows them to explore potential breakthroughs in a mutually beneficial environment. Implementing novel methods and solutions requires a different approach, one focused on operational efficiency and compliance, rather than experimentation. It is also crucial to recognize that different people excel at different stages of the process. Some individuals thrive in the innovation phase, where flexibility and trial-and-error approaches are key. Others are better suited to perform structured, detailed work implementing established solutions. Attempting to blend these mindsets can lead to frustrations as the focus shifts from creative exploration to a more rigid, professionalized implementation. Understanding these distinctions and assigning the right people to the right phases can help maintain momentum and preserve the power of innovation while ensuring successful implementation.

### Conclusions

Multistakeholder initiatives like the medEmotion project highlight the complexity of managing diverse roles, expectations, and goals. Stakeholders are rarely confined to a single role, and roles themselves are often shared among multiple individuals, which can complicate communication and decision-making. We introduce personas to clarify these different roles. Further, it is essential to communicate through full end-to-end use cases, ensuring that all parties understand how the platform functions within the broader ecosystem. In response, we introduce demonstrators to communicate technical requirements efficiently. The project highlighted the importance of building upon existing technical advancements rather than reinventing the wheel. Despite the availability of proven solutions, there was often a reluctance to fully embrace these innovations. This hesitation, driven by a desire to retain ownership of specific platforms or initiatives, leads to fragmented efforts and ultimately diminishes the overall impact. Collaboration, when done openly and transparently, benefits everyone involved, teamwork is crucial to achieving shared success. While technical sandboxes facilitate communication and experimentation, translating these ideas into real-world applications can be a slow and frustrating experience. The journey from concept to implementation requires patience and ongoing commitment. Ultimately, the lessons learned from medEmotion, building on existing successes, fostering collaboration, and maintaining a clear vision, lay the groundwork for more effective and sustainable initiatives in the future.

## Abbreviations

CDM: common data model
FHIR: Fast Healthcare Interoperability Resources
GDPR: General Data Protection Regulation
HIS: hospital information system
HIPAA: Health Insurance Portability and Accountability Act
HPC: high-performance computing
IP: intellectual property
MSDC: multiple sclerosis data connect
OHDSI: Observational Health Data Sciences and Informatics
OMOP: Observational Medical Outcomes Partnership
